# Personals

**Published:** 1918-01

**Authors:** 


### PERSONAL

Announcement of removal, travel, and other matters of interest are requested. Please report errors in the listing of any physician in the State and other directories, that we may co-operate with the State Society in securing a correct list.

---

Dr. C. L. Randall of Franklinville left for Salt Lake City on December 17 to fill a vacancy in the United States Indian reservation department. He expects to be gone till next May.

Dr. Robert C. Kenner, Editor of the Therapeutic Record of Louisville, Ky., spent the first week of December in Buffalo.

Dr. J. W. Grosvenor of Buffalo has returned from a visit in Philadelphia, Pa.

Dr. John J. Hanavan of East Aurora sustained a fractured arm in an auto collision.

Dr. E. E. Hummel, formerly of Darien Centre, N. Y., is now associated with Dr. George L. Brown, and will limit his practice to diseases of the rectum.

Health Officers of Western New York who have been taking a course at the University of Buffalo, gave a dinner on Nov. 21st at the Hotel Statler in honor of Dr. Wm. G. Bissell, city bacteriologist. For six weeks they attended lectures by Dr. Bissell at the city laboratories of the health department in William Street. Those at the dinner were: T. P. Barnard, North Tonawanda; Thos. E. Fleming, Gardenville; F. A. Helwig, Akron; Thos. A. Kerr, Lewiston; Willard B. Jolls, Orchard Park; Chas. Kelley, Franklinville; Myron M. Metz, Williamsville; J. W. Crews, Pittsford; J. H. Leary, Rush; Lester N. Lougee, Marilla; A. W. Wagner, Buffalo; A. B. Harding, Castile; H. C. Mann, Holland; J. J. Redmond, Fillmore; F. A. Watters, Lockport; A. E. Brennan, Erie; W. H. Baker, Williamsville; and S. G. Hermance, Clarkson.

Dr. George E. Fell of Chicago, Ill., announces a series of addresses to be given before the lodges of the Masonic fraternity of the State of Illinois.

Dr. Alfred H. Noehren of East Utica street, Buffalo, has returned from Rochester, Minn.

